# Supplementary material for: FERN – a Java framework for stochastic simulation and evaluation of reaction networks
Source: BMC Bioinformatics. 2008 Aug 29;9:356. doi: 10.1186/1471-2105-9-356 (PMC2553347; doi:10.1186/1471-2105-9-356)
Supplement: Additional file 1 — FERN distribution, Version 1.3. This archive contains the FERN source code and binaries as well as documentation and example models in FernML and SBML. [file 1471-2105-9-356-S1.zip › fern/doc/javadoc/fern/simulation/algorithm/GillespieSimple.html]

GillespieSimple


---


|  |  |  |
| --- | --- | --- |
| **Overview** \| **Package** \| **Class** \| **Use** \| **Tree** \| **Deprecated** \| **Index** \| **Help** | |  |
| **PREV CLASS**   **NEXT CLASS** | **FRAMES**    **NO FRAMES**     **All Classes** |  |
| SUMMARY: NESTED \| FIELD \| CONSTR \| METHOD | DETAIL: FIELD \| CONSTR \| METHOD |  |


---


## fern.simulation.algorithm Class GillespieSimple

```
java.lang.Object
  fern.simulation.Simulator
      fern.simulation.algorithm.GillespieSimple
```

---

``` public class GillespieSimple extends Simulator ```

Implementation of Gillespie's Direct method. It is a simple
Monte-Carlo algorithm which draws from a from Gillspie derived distribution
a reaction that will fire and a time at which the reaction will fire.

For reference see Daniel T. Gillespie., A General Method for Numerically Simulating
the Stochastic Time Evolution of Coupled Chemical Reactions, J.Comp.Phys. 22, 403 (1976)

**Author:**
:   Florian Erhard

---

**Nested Class Summary**

**Nested classes/interfaces inherited from class fern.simulation.Simulator**

`Simulator.FireType`


**Field Summary**

**Fields inherited from class fern.simulation.Simulator**

`a, stochastics, t`


**Constructor Summary**

`GillespieSimple(Network net)`


| **Method Summary** | |
| --- | --- |
| `protected  double` | `directMCTau(double sum)`             obtains a random (but following a specific distribution) timestep as described by the direct method in chapter 5A page 417ff |
| `String` | `getName()`             Gets the name of the algorithm. |
| `void` | `performStep(SimulationController control)`             Performs one simulation step. |
| `void` | `reinitialize()`             Reset propensities when a event has been executed. |

**Methods inherited from class fern.simulation.Simulator**

`addObserver, fireReaction, fireReaction, getAmount, getAmountManager, getNet, getNextThetaEvent, getPropensity, getPropensityCalculator, getTime, getVolume, initialize, isInterpolateTheta, registerNewTheta, setAmount, setInterpolateTheta, setVolume, start, start, thetaEvent`

**Methods inherited from class java.lang.Object**

`clone, equals, finalize, getClass, hashCode, notify, notifyAll, toString, wait, wait, wait`

**Constructor Detail**

### GillespieSimple

```
public GillespieSimple(Network net)
```


**Method Detail**

### reinitialize

```
public void reinitialize()
```

:   **Description copied from class: `Simulator`**
:   Reset propensities when a event has been executed.

    :   **Specified by:**: `reinitialize` in class `Simulator`

---


### performStep

```
public void performStep(SimulationController control)
```

:   **Description copied from class: `Simulator`**
:   Performs one simulation step. Between steps the terminating condition is checked.
    The simulators controller is passed, if an extending class wants to check it
    within one step. Implementing algorithms cannot be sure that the propensities
    are correct at the beginning of a step (e.g. if the volume changed). They should
    override `Simulator.setAmount(int, long)` and `Simulator.setVolume(double)`
    if they need correct values!

    :   **Specified by:**: `performStep` in class `Simulator`
    :   **Parameters:**: `control` - the simulators controller

---


### directMCTau

```
protected double directMCTau(double sum)
```

:   obtains a random (but following a specific distribution) timestep as described by the
    direct method in chapter 5A page 417ff

    :   **Parameters:**: `sum` - sum of the propensities **Returns:**: tau

---


### getName

```
public String getName()
```

:   **Description copied from class: `Simulator`**
:   Gets the name of the algorithm.

    :   **Specified by:**: `getName` in class `Simulator`
    :   **Returns:**: name of the algorithm


---


|  |  |  |
| --- | --- | --- |
| **Overview** \| **Package** \| **Class** \| **Use** \| **Tree** \| **Deprecated** \| **Index** \| **Help** | |  |
| **PREV CLASS**   **NEXT CLASS** | **FRAMES**    **NO FRAMES**     **All Classes** |  |
| SUMMARY: NESTED \| FIELD \| CONSTR \| METHOD | DETAIL: FIELD \| CONSTR \| METHOD |  |


---
